# Dichloroacetate Enhances Adriamycin-Induced Hepatoma Cell Toxicity *In Vitro* and *In Vivo* by Increasing Reactive Oxygen Species Levels

**DOI:** 10.1371/journal.pone.0092962

**Published:** 2014-04-11

**Authors:** Yunhai Dai, Xiaopeng Xiong, Gang Huang, Jianjun Liu, Shile Sheng, Hongjian Wang, Wenxin Qin

**Affiliations:** 1 Department of Nuclear Medicine, Renji Hospital, Shanghai Jiaotong University School of Medicine, Shanghai, China; 2 Institute of Health Sciences, Shanghai Jiao Tong University School of Medicine (SJTUSM) & Shanghai Institutes for Biological Sciences (SIBS), Chinese Academy of Sciences (CAS), Shanghai, China; 3 Department of Gastroenterology, Sixth People's Hospital, Shanghai Jiaotong University School of Medicine, Shanghai, China; 4 National Laboratory for Oncogenes and Related Genes, WHO Collaborating Center for Research on Cancer, Shanghai Cancer Institute, Shanghai Jiao Tong University, Shanghai, China; Columbia University, United States of America

## Abstract

A unique bioenergetic feature of cancer, aerobic glycolysis is considered an attractive therapeutic target for cancer therapy. Recently, dichloroacetate (DCA), a small-molecule metabolic modulator, was shown to reverse the glycolytic phenotype, induce reactive oxygen species (ROS) generation, and trigger apoptosis in various tumor cells. In this work, the capacity of DCA to enhance Adriamycin (ADM) efficacy in hepatoma cells by modulating glucose metabolism and redox status was evaluated. Two human hepatoma (HCC-LM_3_ and SMMC-7721) and a normal liver (LO_2_) cell lines were treated with DCA or ADM alone, or in combination. Exposure of hepatoma cells to DCA/ADM combination resulted in significantly decreased cell viability and increased percentage of apoptotic cells as well as intracellular ROS levels, in comparison with treatment with DCA or ADM alone. However, simultaneous treatment with the thiol antioxidant N-acetylcysteine (NAC, 10 mmol/L) reduced the elevated ROS levels and protected hepatoma cells from the cytotoxic effects of DCA/ADM combination. L-buthionine-[S,R]-sulfoximine, an inhibitor of glutathione synthesis, enhanced hepatoma cell sensitivity to DCA/ADM combination. Interestingly, treatment with DCA/ADM combination did not significantly increase cytotoxicity in normal hepatocytes in comparison with the drugs administered individually. Finally, DCA reduced tumor growth and enhanced ADM efficacy on HCC-LM_3_ hepatoma in mice. Overall, our data suggest that DCA enhances ADM cytotoxicity in hepatoma cells by increasing intracellular ROS levels and provide a strong biochemical rationale for the use of DCA in combination with ADM for treatment of hepatoma.

## Introduction

In the 1920s, Otto Warburg described the high glycolysis rate of cancer cells when compared with normal cells, even in the presence of oxygen [1]. This phenomenon, known as Warburg effect and also termed aerobic glycolysis, describes the ability of cancer cells to increase glucose uptake and convert most of the pyruvate to lactate, reducing the mitochondrial pyruvate pool. This metabolic feature, frequently observed in cancer cells of various tissue origins, constitutes a serious target for cancer prevention and therapeutic strategies. Indeed, ongoing studies are investigating possible ways to exploit or interrupt tumor glycolytic metabolism in cancer cells.

Several small molecules have been described with various degrees of anticancer activity *in vitro* and *in vivo* [2]–[5]. However, many existing glycolysis inhibitors (such as 2-deoxy-D-glucose (2-DG) and 3-bromopyruvate (3-BrPA) do not seem to be effective tumor growth inhibitors when used as monotherapy, since the required dose to achieve clinical efficacy is associated with toxicity. Nevertheless, some of these compounds are currently being explored as agents to enhance the therapeutic effects of radiation therapy or cytotoxic drugs such as Adriamycin and Paclitaxel [6]–[13].

Recently, dichloroacetate (DCA) was shown to reverse the glycolytic phenotype and induce cell injury in a number of cancer cell lines of breast, prostate, lung, medullary thyroid, and endometrial cancers, as well as myeloma and glioblastoma multiforme [14]–[20]. Pyruvate dehydrogenase (PDH) is a key regulator in cellular metabolism and is in turn inhibited by pyruvate dehydrogenase kinase (PDK). DCA, a synthetic PDK inhibitor, was shown to reverse glycolysis to oxidative phosphorylation through PDH activation. This metabolism shift decreases mitochondrial membrane potential hyperpolarization, opens mitochondrial transition pores, and allows for elevated generation of reactive oxygen species (ROS) and translocation of cytochrome C from mitochondria to cytoplasm, subsequently inducing apoptosis through caspase activation [14]. Currently, a few reports on a combination of DCA and radiation therapy or cytotoxic drugs are available. For instance, Ayyanathan *et al.* [21] studied the effect of DCA combination with Sulindac, a non-steroidal anti-inflammatory drug with documented anticancer activities. In addition, Sanchez *et al*. [17] demonstrated that DCA increases sensitivity of myeloma cells to Bortezomib.

There are limited chemotherapy options for hepatocellular carcinoma (HCC), with Adriamycin (ADM) likely the most widely used drug. Indeed, a 16% response was documented in HCC patients receiving ADM as monotherapy [22]. Combining ADM with a targeted therapeutic approach may, therefore, provide additional treatment options. ADM use in cancer therapy is limited by its cytotoxicity to cardiomyocytes [23] and hepatocytes [24]. The clinically relevant dose of ADM for use *in vitro* has been shown to be 0.5 µmol/L, calculated from the maximum dose typically used in cancer treatment [25]. In the present study, we investigated the anti-tumor efficacy of DCA in HCC *in vitro* and *in vivo*, and evaluated the efficacy of DCA and ADM in combination.

## Materials and Methods

### Cells and cell culture

HCC-LM_3_ and LO_2_ cell lines were purchased from the Liver Cancer Institute, Zhongshan Hospital at Fudan University, and SMMC-7721 cells were obtained from the Chinese Type Culture Collection (Shanghai). All cells were cultured in a humidified environment with 5% CO2 at 37°C in DMEM containing 25 mmol/L glucose supplemented with 10% fetal bovine serum (FBS; Hyclone, Logan, UT).

### Drug treatment

DCA, N-acetylcysteine (NAC), and L-buthionine-[S,R]-sulfoximine (BSO) were obtained from Sigma Chemical Co. (St. Louis, MO). ADM was obtained from Aladdin reagent Inc. (Shanghai). For treatments, drug samples were added directly to complete cell culture media on cells to achieve final concentrations of 10–20 mmol/L (DCA), 0.5 µmol/L (ADM), 10 mmol/L (NAC), and 1.0 mmol/L (BSO). Stock solutions were diluted in PBS except NAC for which 1 mol/L sodium bicarbonate (pH 7.0) was used.

### Coefficient of drug interaction

The coefficient of drug interaction (CDI) was used to analyze the effects of drug combination as previously described [26], and derived as follows: CDI  =  AB/(A×B), where AB is the absorbance ratio of the combination group to control group; A or B is the ratio obtained after treatment with a single agent. Thus, CDI values <1,  = 1 and >1 indicate that drugs are synergistic, additive or antagonistic, respectively. CDI values of less than 0.7 indicate significant synergistic effects. All experiments were performed for at least 3 times.

### Cell viability assay

Cell viability was determined using the Cell Counting Kit-8 (CCK-8; Dojindo, Kumamoto, Japan) assay according to the manufacturer's guide. Briefly, cells were seeded into 96-well plates (10,000 cells per well) and incubated overnight. Then, the cells were treated with various drug samples for 48 h, whereafter CCK-8 solution was added to the cell culture medium to a final concentration of 10 µL/ µL. After further incubation for 2 h at 37°C, cell viability was determined spectrophotometrically at 450 nm (with reference reading at 650 nm) on a microplate reader. Experiments were performed in triplicate, and all tests repeated at least 3 times.

### Apoptosis assay

Apoptotic rates were analyzed by flow cytometry using the Annexin V-FITC/propidium iodide (PI) kit (Merck4 Biosciences). Briefly, cells were seeded into 6-well plates overnight and exposed to different drug samples. After 24 h incubation, cells were washed twice with PBS and resuspended in 1 ml binding buffer. Finally, a total of 500 µl cell suspension was incubated in presence of µl Annexin V-FITC and 10 µl PI for 10 min at room temperature in the dark and flow cytometry performed on a BD FACScan flow cytometer (BD Bioscience). Data were acquired and analyzed using the CellQuest Pro software (BD).

### Glucose consumption measurements

Glucose contents were measured using a glucose assay kit (Sigma-Aldrich, St Louis, MO, USA) according to the manufacturer's instructions. Briefly, 4×10^5^ cells were seeded into 6-well plates and incubated overnight. Then, cells were cultured in complete medium (10% fetal calf serum (FCS), 25 mmol/L glucose) in presence of various test articles for 48 h. After treatment, media were collected and stored at −20°C until use, and cell counts were performed using the Z2 Coulter particle count and size analyzer (Beckman Coulter). The consumption of glucose was determined and expressed as µmol/L/10^6^ cells/24 h. All tests were performed in triplicate and experiments repeated at least 3 times.

### Assessment of lactate production

Lactate production was measured as described by Rodríguez-Enríquez *et al*. [27]. Briefly, 4×10^5^ cells were seeded into 6-well plates and incubated overnight. Then, cells were cultured in complete medium (10% FCS, 25 mmol/L glucose) containing different concentrations of test articles for 48 h. After treatment, cells were washed three times with pre-warmed modified Krebs–Ringer medium. Next, cells were incubated in 1 ml modified Krebs– Ringer medium (25 mmol/L glucose) at 37°C for 30 min. Supernatants were collected for lactate measurements using an enzymatically coupled lactate detection reagent (Sigma) according to the manufacturer's instructions. Cell counts were performed as described above and lactate production calculated and expressed as nmol/L/10^6^ cells/30 min. All tests were performed in triplicate and experiments repeated at least 3 times.

### Reactive oxygen species (ROS) measurements

ROS levels were detected using CM-H2DCFDA (Molecular Probes, NY, USA), according to the manufacturer's instructions. Cells (1×10^5^) were seeded in 1 ml complete medium in 12-well plates and allowed to adhere overnight. Then, cells were treated with various drug samples for 12 h and culture media replaced by 0.4 ml freshly prepared CM-H2DCFDA solution. After 30 min incubation at 37°C, cells were harvested by trypsinization and resuspended in PBS buffer. Finally, fluorescence of a 10^4^ cell suspension was measured using a microplate reader (excitation 485 nm, emission 520 nm).

### *In vivo* Effect of DCA and ADM in HCC-LM_3_ hepatoma Xenograft

5×10^6^ HCC-LM_3_ hepatoma cells were s.c. injected into the right flank regions of 5-6 week old male nude mice (BalB/c nu^+^/nu^+^) obtained from the Shanghai Cancer Institute. When tumors were approximately 200 mm^3^ in size, mice were randomly divided into four groups of 8 set to receive saline (control), DCA alone, ADM alone, and DCA/ADM combination (DCA+ADM). DCA (0.75 g/L) was added to drinking water for mice in DCA alone and DCA+ADM groups according to the method of Bonnet et al [28]. On day 1, mice in ADM and DCA+ADM groups were intravenously administered 0.2 ml ADM at 0.6 mg/ml (6 mg/kg) and this treatment was repeated once weekly for a total of three doses (18 mg/kg). Mice were weighed and tumor sizes measured using a caliper three times weekly and tumor volumes derived as W×L^2^/2, where W and L represent width and length, respectively. 5 weeks after treatment, mice were sacrificed and weighed, and tumors were excised, weighed and analyzed histologically. All experimental procedures were approved by the Animal Use Committee of the Shanghai Cancer Institute.

### Statistical analyses

Statistical analyses were carried out using the GraphPad (GraphPad Software, Inc., San Diego, CA) and SPSS (SPSS Inc., Chicago, IL) software. Each experiment was performed in triplicate, and all tests repeated at least 3 times. Differences between groups were analyzed by one-way ANOVA with Bonferroni (LSD) post-tests. Error bars represent standard deviations (SD) and differences were considered statistical significant if p<0.05.

## Results

### Treatment with DCA/ADM combination enhanced cytotoxicity in hepatoma cells

**Figure 1:**
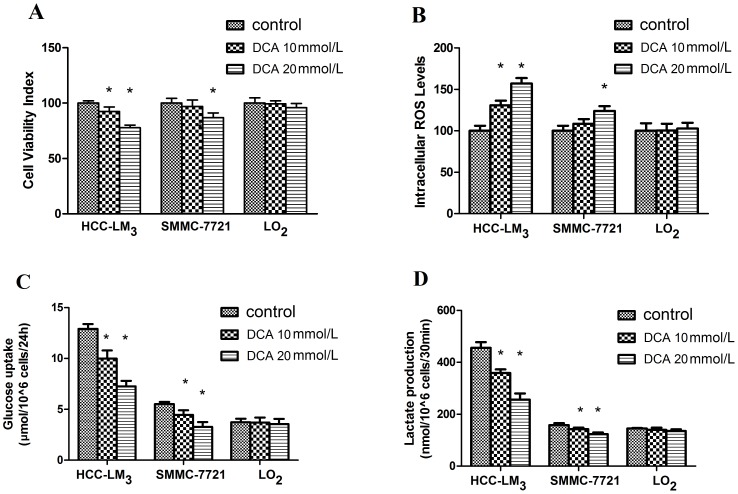
Effect of DCA on cell viability, intracellular ROS levels and glucose metabolism in hepatoma cells after 48/L DCA. (A) Cell viability was determined as described above. 20 mmol/L DCA treatment decreased viability of the 2 hepatoma cell lines but not the normal hepatocyte cell line LO_2_. DCA at 10 mmol/L reduced cell viability significantly only in HCC-LM_3_ cells; (B) 20 mmol/L DCA treatment resulted in elevated intracellular ROS generation in hepatoma cell lines but not LO_2_. DCA at 10 mmol/L induced ROS level increase only in HCC-LM_3_ cells; (C) DCA at 10 and 20 mmol/L inhibited glucose uptake in both hepatoma cell lines but not LO_2_; (D) DCA at 10 and 20 mmol/L inhibited lactate production in both hepatoma cell lines but not LO_2_. * indicates statistical significance versus control cells (p<0.05). Each experiment was performed in triplicate, and all tests were repeated at least 3 times to provide this data.

**Figure 2:**
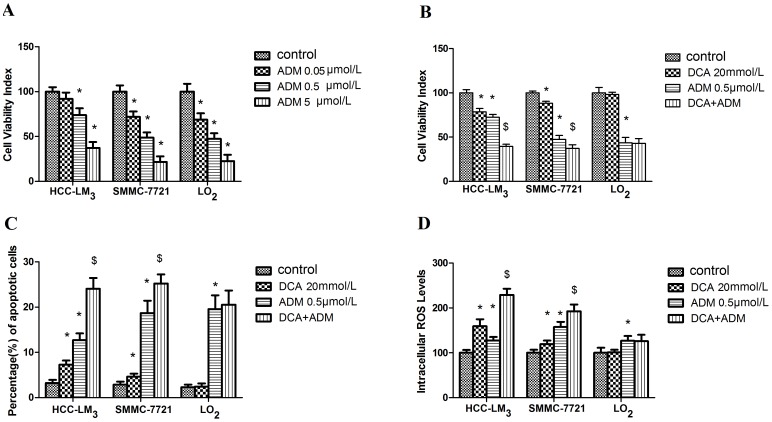
Effect of DCA (20 mmol/L), ADM (0.5 µmol/L), and DCA/ADM combination (20 mmol/L DCA and 0.5 µmol/L ADM) on cell viability, intracellular ROS levels and induction of apoptosis in hepatoma cells. (A) Cell viability test of different concentrations of ADM on the three cell lines. (B) Treatment with DCA/ADM combination significantly decreased cell viability in the hepatoma cells in comparison with control and ADM treatments but not in the LO_2_ cells; (C) Treatment with DCA/ADM combination significantly increased apoptotic cell percentage in HCC-LM_3_ and SMMC-7721 cells compared with controls and ADM treatment but the combination did not increase the apoptotic cell percentage compared to ADM in the normal LO_2_ cells; (D) Treatment with DCA/ADM combination resulted in a significant increase in intracellular ROS levels in the hepatoma cells compared with controls, and cells treated with ADM alone but not in the LO_2_ cells. * and $ indicate statistical significance versus control cells and cells treated with ADM alone, respectively (p<0.05). Each experiment was performed in triplicate, and all tests were repeated at least 3 times to provide this data.

Our preliminary data showed that DCA at 20 mmol/L decreased the viability of both hepatoma cell lines but not the LO_2_ cell line of normal hepatocytes. 10 mmol/L DCA reduced cell viability significantly only in HCC-LM_3_ cells. 20 mmol/L DCA treatment resulted in elevated intracellular ROS generation in hepatoma cell lines but not in the LO_2_ cell line. DCA at 10 mmol/L induced ROS level to increase only in HCC-LM_3_ cells. 10 and 20 mmol/L DCA inhibited glucose uptake in both hepatoma cell lines but not LO_2_. 10 and 20 mmol/L DCA inhibited lactate production in both hepatoma cell lines but not in the LO_2_ cell line. So as the 20 mmol/L level was effective in modulating glucose metabolism and increasing ROS generation in the hepatoma cells (Fig. 1), 20 mmol/L DCA was chosen for subsequent experiments. 0.5 µmol/L ADM, a clinically relevant dose within achievable plasma levels in HCC patients during ADM therapy [27] that also significantly decreased cell viability of all the cell lines (Fig. 2A) was selected as the best treatment level for ADM.

Cell viability was determined using the Cell Counting Kit-8 assay. As shown in Fig. 2B, viability of HCC-LM_3_ and SMMC-7721 hepatoma cells was reduced after treatment with DCA and ADM alone or in combination, after the 48 h exposure. Interestingly, treatment with the combination of 20 mmol/L DCA and 0.5 µmol/L ADM resulted in a significant decrease in cell viability in comparison with DCA and ADM individual treatments, with 37.11%, 88.82%, and 47.32% live SMMC-7721 cells observed after exposure to DCA/ADM, DCA and ADM, respectively. Likewise, only 39.50% live HCC-LM_3_ cells were measured after treatment with the combination, a significance difference compared with 78.59% and 72.35% obtained for DCA and ADM, respectively. These findings suggest at least an additive and possibly a synergistic effect of DCA and ADM.

CDI is a parameter that is widely used to analyze the nature of drug interactions. We obtained CDI values of 0.69 and 0.89 in HCC-LM_3_ and SMMC-7721 hepatoma cells, respectively, indicating a significant synergism of these drugs.

Importantly, treatment with 20 mmol/L DCA did not significantly decrease cell viability in normal hepatocyte cells, LO_2_. In addition, the combination did not result in decreased cell viability in comparison with ADM alone (42.89% versus 43.62%, respectively, p>0.05).

Next, the effects of DCA, ADM, and DCA/ADM combination on apoptosis induction were evaluated by flow cytometry after Annexin-V-FITC/PI cell staining in the cell lines. We found that treatment with DCA/ADM combination resulted in significant increase in apoptotic cell percentage in comparison with control cells and cells exposed to DCA or ADM individually in the hepatoma cell lines (Fig. 2C). While in the LO_2_ normal hepatocyte cells, the combination did not result in significant increase in apoptotic cell percentage in comparison with ADM alone (Fig. 2C).

### DCA/ADM combination induced a significant increase in hepatoma cell ROS levels

After individual treatment of HCC-LM_3_ with 20 mmol/L DCA and 0.5 µmol/L ADM, relative ROS levels of 159.45% and 127.28% were obtained, respectively. These values increased when the two drugs were combined to 228.99%. Similar results were observed for SMMC-7721 with 192.36%, 119.03%, and 157.63% for the combination, DCA, and ADM, respectively (Fig. 2D). These data suggest a higher capacity of the combination to induce intracellular ROS generation in comparison with individual drugs. Interestingly, 20 mmol/L DCA treatment caused no significant increase in LO_2_ intracellular ROS levels. In addition, intracellular ROS levels were not higher in these normal hepatocytes after treatment with the combination in comparison with ADM alone (combination: 124.00%; ADM: 122.85%; p>0.05).

### NAC inhibits increased ROS levels and compromises the enhanced cytotoxicity of the DCA/ADM combination

To investigate the possible involvement of intracellular ROS level increase in the enhanced cytotoxicity observed with the drug combination, HCC-LM_3_ and SMMC-7721 cells were treated with the thiol antioxidant NAC (10 mmol/L) for 1 h before and during drug exposure.

**Figure 3:**
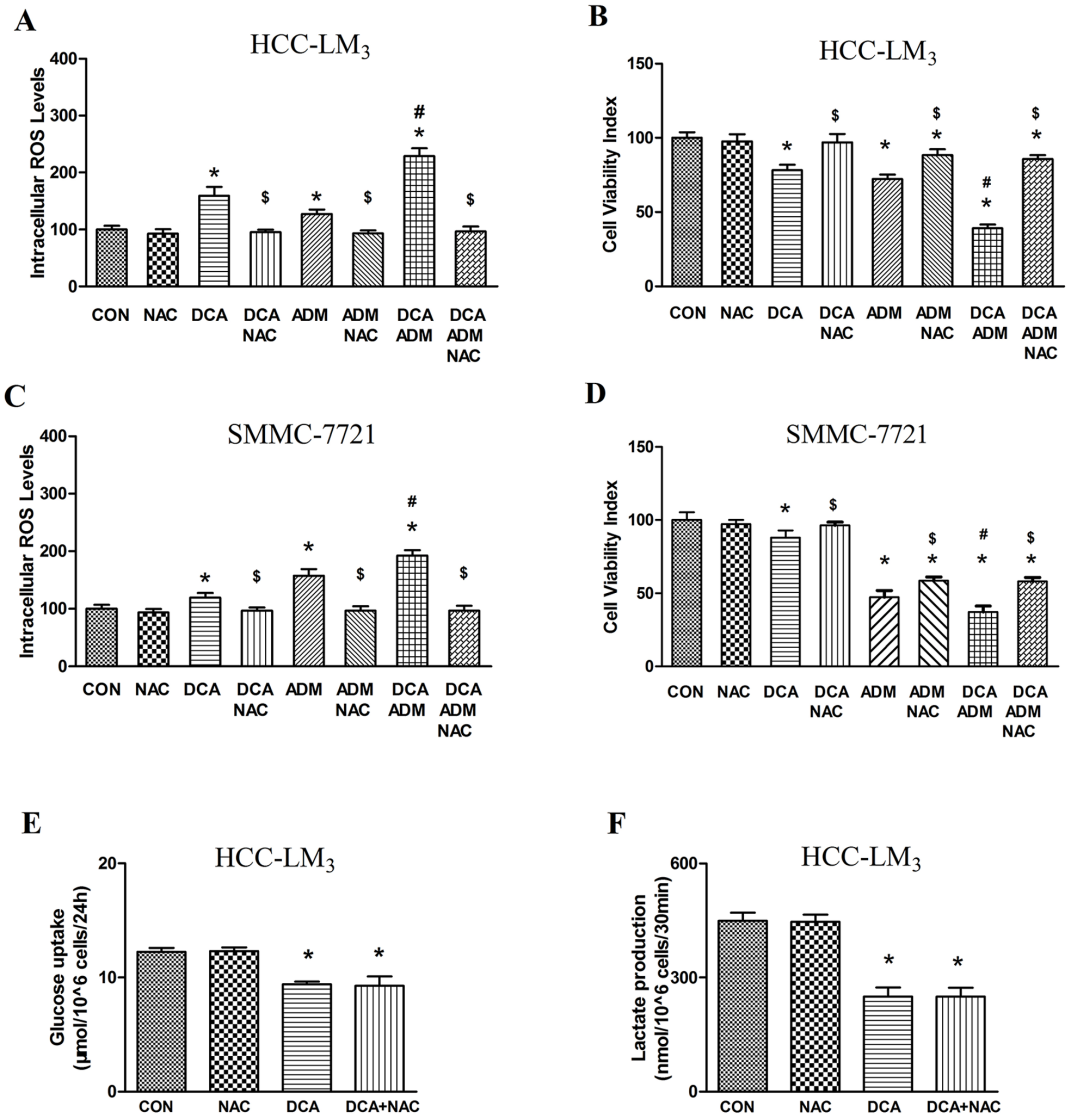
ROS inhibitor NAC compromises cytotoxicity of ADM/DAC treatment in HCC-LM_3_ and SMMC-7721 cell lines but does not impact glucose metabolism which is depressed by DAC. (A) Simultaneous treatment with 10 mmol/L NAC restored ROS to basal levels in HCC-LM_3_ cells; (B) Simultaneous treatment with 10 mmol/L NAC significantly inhibited the enhanced cytotoxicity observed with DCA/ADM combination in HCC-LM_3_ cells; (C) Simultaneous treatment with 10 mmol/L NAC restored ROS to basal levels in SMMC-7721 cells; (D) Simultaneous treatment with 10 mmol/L NAC significantly inhibited the enhanced cytotoxicity observed with DCA/ADM combination in SMMC-7721 cells. *, $, and # indicate statistical significance versus control cells, corresponding treatment without NAC, and DCA+ADM versus ADM alone, respectively (p<0.05). (E) Treatment with 20 mmol/L DCA significantly decreased glucose consumption in HCC-LM_3_ cells with or without 10 mmol/L NAC; (F) Treatment with 20 mmol/L DCA significantly decreased lactate production in HCC-LM_3_ cells with or without 10 mmol/L NAC. * indicates statistical significance versus control cells (p<0.05). Each experiment was performed in triplicate, and all tests were repeated at least 3 times to provide this data.

Treatment with NAC alone showed no significant effect on intracellular ROS levels. However, coincubation of 10 mmol/L NAC with DCA and/or ADM in the HCC-LM_3_ cells resulted in significantly lower intracellular ROS levels compared with the respective treatment groups without NAC, indicating that the oxidative stress induced by DCA and/or ADM was attenuated with exposure to NAC (Fig. 3A).

Similar data were obtained for cytotoxicity as demonstrated by the cell viability assays in HCC-LM_3_ cells. Coincubation with 10 mmol/L NAC inhibited DCA induced cytotoxicity and partially but significantly inhibited ADM cell killing in HCC-LM_3_. In addition, we found that exposure to 10 mmol/L NAC significantly compromised DCA/ADM combination induced cytotoxicity in HCC-LM_3_ cells (DCA+ADM+NAC:85.71%; ADM+NAC:88.27%, p>0.05. Fig. 3B).

The coincubation of 10 mmol/L NAC with DCA and/or ADM experiments in SMMC-7721 showed similar results (Fig. 3C and D).

Taken together, these data suggest that the enhanced cytotoxic effects of DCA/ADM combination resulted from their capacity to induce oxidative stress.

We also found, however, that 10 mmol/L NAC had no effect on the decreased glucose metabolism and lactate production seen with 20 mmol/L DCA in HCC-LM_3_ (Fig. 3E and F).

### BSO augments increased ROS levels and boosts the enhanced cytotoxicity of the DCA/ADM combination

**Figure 4:**
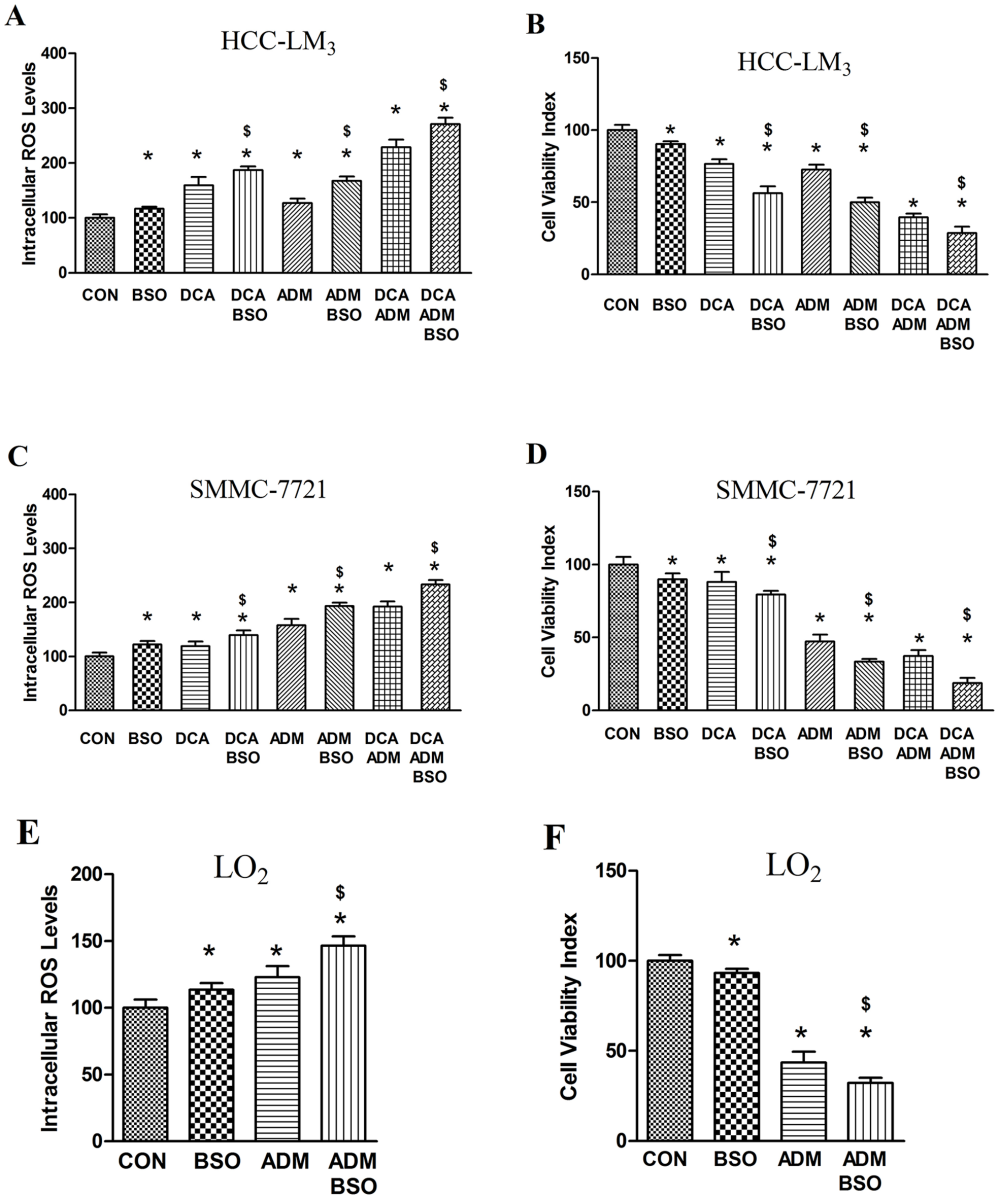
BSO has a similar additive cytotoxic effect on ADM as that seen with DAC in liver cancer cell lines but in normal hepatic cell line LO_2_, BSO also has additive cytotoxic effect on ADM while DAC does not. (A) Simultaneous treatment with 1 mmol/L BSO further enhanced the increase in intracellular ROS levels in HCC-LM_3_ cells; (B) Simultaneous treatment with 1 mmol/L BSO further enhanced the increase in cytotoxicity in HCC-LM_3_ cells; (C) Simultaneous treatment with 1 mmol/L BSO further enhanced the increase in intracellular ROS levels in SMMC-7721 cells; (D) Simultaneous treatment with 1 mmol/L BSO further enhanced the increase in cytotoxicity in SMMC-7721 cells. * and $ indicate statistical significance versus control cells and corresponding treatments without BSO, respectively (p<0.05). (E) Treatment with the combination of 1 mmol/L BSO and 0.5 µmol/L ADM shows further increase of intracellular ROS levels in LO_2_ normal hepatocyte cell lines; (F) Treatment with the combination of 1 mmol/L BSO and 0.5 µmol/L ADM shows enhanced cytotoxicity in LO_2_ normal hepatocyte cell lines. * and $ indicate statistical significance versus control cells and cells treated with ADM alone, respectively (p<0.05). Each experiment was performed in triplicate, and all tests were repeated at least 3 times to provide this data.

To determine if glutathione (GSH) depletion would enhance drug induced cytotoxicity and oxidative stress, HCC-LM_3_ cells were treated with 1 mmol/L BSO for 1 h before and during DCA and/or ADM exposure. As shown in Fig. 4A, exposure to BSO resulted in higher increases in intracellular ROS levels compared with corresponding treatment groups without BSO.

As expected, BSO further increased HCC-LM_3_ cytotoxicity of DCA and ADM individual treatments as well as DCA/ADM combination (Fig. 4B). SMMC-7721 cells were also treated with 1 mmol/L BSO for 1 h before and during DCA and/or ADM exposure, and similar data were obtained (Fig. 4C, and D). These results indicate that BSO effectively decreased intracellular GSH and increased oxidative stress when coincubated with DCA and/or ADM, confirming that increases in ROS levels contribute to DCA/ADM combination induced cytotoxicity.

Unlike the DCA/ADM combination which shows no significant enhanced cytotoxicity in LO_2_ normal liver cells, treatment with BSO/ADM in combination resulted in intracellular ROS level increase and enhanced cytotoxicity in LO_2_ cells (Fig. 4E and F).

### DCA enhances ADM induced cytotoxicity in HCC-LM_3_ hepatoma cells *in vivo*

The effects of DCA and ADM were studied on nude athymic mice implanted subcutaneously with HCC-LM_3_ cells. When tumors were approximately 200 mm^3^ in size, mice were randomly divided into four groups (n = 8 in each group) and treated with control vehicle, DCA, ADM, and DCA/ADM combination, as described in the experimental section. We monitored continuously the situation of the tumor bearing mice including survival time, body weight, food intake, water intake, activity, etc, until anatomy. No mouse died during the research and there was no significant difference of these indicators among the groups.

**Figure 5:**
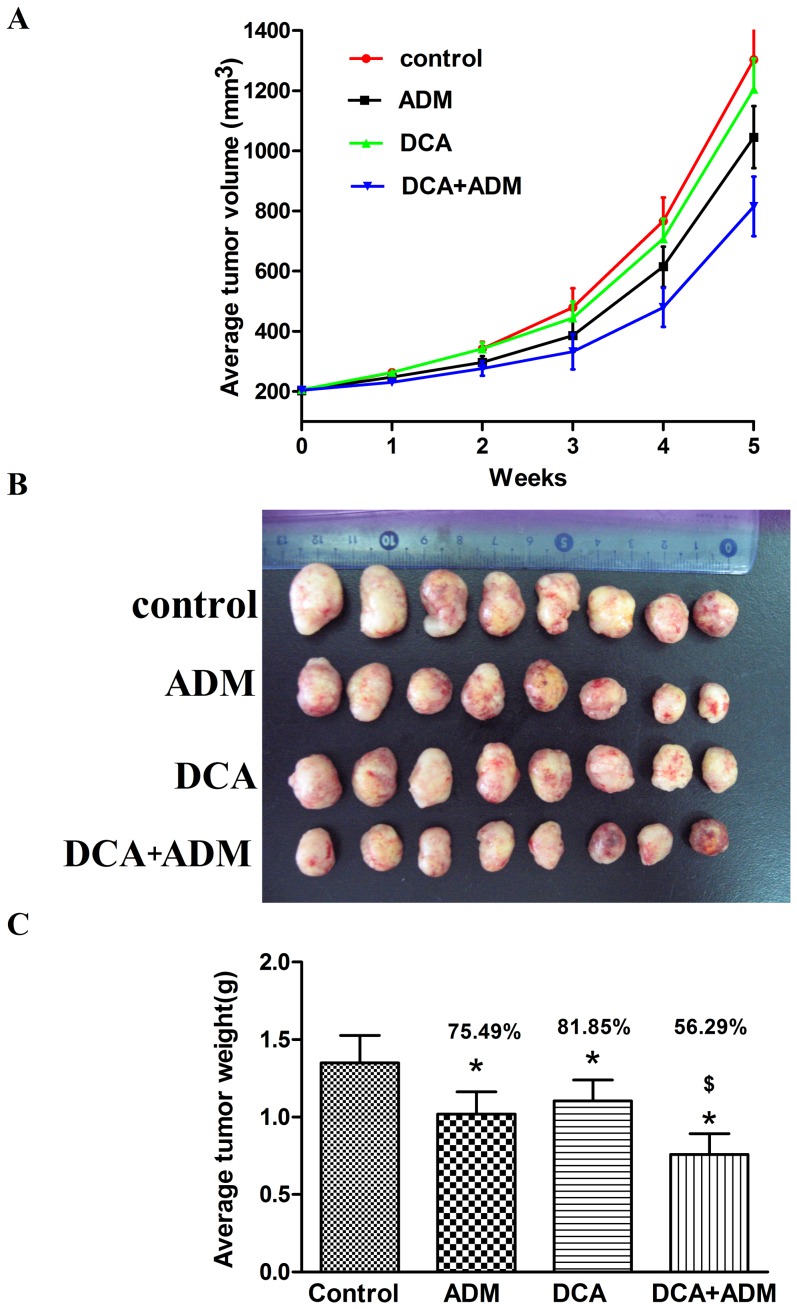
Effect of DCA and ADM alone and in combination on growth of HCC-LM_3_ xenografts in nude mice (n = 8 in each group). (A) Time course of tumor volumes. Treatment with DCA/ADM combination resulted in enhanced efficacy compared with either agent alone. (B) Images, and (C) tumor weights and inhibition rates of excised tumors show enhanced efficacy of the combined treatment as compared with either agent alone. * and $ indicate statistical significance versus control and groups treated with either agent alone (p<0.05).

As shown in Fig. 5A and B, control group mice rapidly developed tumors with a constant exponential growth, while mice treated with DCA and/or ADM presented significantly reduced tumor sizes. Precisely, tumor volume averaged 1303.49±143.78, 1052.89±123.38, 1204.37±122.60, and 815.25±119.36 mm^3^ after treatment with the vehicle, ADM, DCA, and DCA/ADM combination, respectively. These data clearly show that DCA/ADM combination is more effective in tumor growth inhibition compared with individual drugs. These results were in accordance with the weights of HCC-LM_3_ xenografts obtained at sacrifice. As shown in Fig. 5C, tumor weights averaged 1.35±0.18,1.02±0.14,1.10±0.13,0.76±0.13 g after treatment with the vehicle, ADM, DCA, and DCA/ADM combination, respectively.

## Discussion

Mitochondria are considered the major source of cellular ROS. During oxidative phosphorylation in mitochondria, as electrons are delivered through the respiratory chain, some may escape from the mitochondrial electron transport chain, especially from complexes I and III. These electrons may then react with molecular oxygen to form superoxide which then converts to H_2_O_2_ and other reactive oxygen species [29].

DCA has recently been shown to modulate glucose metabolism in cancer cells, shifting metabolism from glycolysis to glucose oxidation. It was suggested that increased oxidative phosphorylation in mitochondria may result in elevated ROS generation [30]. Indeed, DCA was shown to increase ROS levels in various cancer cell lines. In addition, Stockwin *et al*. [18] reported the selective activity of DCA against cancer cells deficient in the electron transport chain. We found that DCA induces apoptosis of hepatoma cells by promoting oxidative stress (increasing ROS generation), likely through inhibition of mitochondrial PDK and glycolytic phenotype reversion. Interestingly, DCA effects were fully reversed by NAC, further supporting that metabolic oxidative stress was causally related to the cytotoxicity seen in hepatoma cells after treatment with DCA.

Improving therapeutic activity and selectivity is a major goal in the development of new anticancer strategies. Mounting evidence suggests that many cancer cell types show two distinct biochemical alterations including increased aerobic glycolysis and higher ROS levels compared with their non-cancerous counterparts [30]–[32], which can be exploited for therapeutic benefits.

Although cancer cells exhibit increased glycolysis and mainly rely on this pathway, inhibition of glycolysis alone may not be sufficient to effectively kill malignant cells. Indeed, glycolysis inhibitors such as 2-DG, 3-BrPA, and DCA do not show significant effects on tumor growth when used singly (monotherapy) since the high doses required to achieve clinical efficacy are often associated with toxicity [4].

Likewise, although a few agents that either promote ROS generation or interfere with antioxidant enzymes have been found effective in many cancer cases, low clinical response and resistance were also reported [32]. Suggestions have been made to explore the combination of radiotherapy or conventional chemotherapy with agents that inhibit aerobic glycolysis and/or increase ROS levels in cancer cells, as novel anticancer strategy. Interestingly, glucose deprivation, 2-DG and 3-BrPA, which target aerobic glycolysis, were reported to enhance the cytotoxicity of Cisplatin, Adriamycin, and radiotherapy [6]–[13]. Agents promoting ROS generation or abrogating antioxidant systems in cancer cells have also been used to boost conventional cytotoxic anticancer drugs as well as radiation [33]–[35]. Recently, DCA was shown to increase sensitivity of myeloma cells to bortezomib [17]. However, such experiments have not been carried out for hepatocellular carcinoma, a prevalent cancer with treatment challenges.

Our results indicate that DCA can effectively reverse the glycolytic phenotype and increase ROS generation in hepatoma cells, suggesting its potential as sensitizer for radiotherapy or conventional chemotherapy. Indeed, DCA significantly enhanced ADM induced cytotoxicity in hepatoma cells both *in vitro* and *in vivo* as shown above. For the *in vivo* experiments DCA was added to the drinking water of the treated rats following an effective method of drug administration [28]. We are relying upon the average results from the eight animals treated and that under most conditions the mice will drink the water to ensure that the animals receive a suitable dose of the drug. An important fact worth noting is that although ADM is cytotoxic to normal cells as well as hepatoma cells, DCA/ADM in combination in the LO_2_ cells did not enhance the ADM induced cytotoxicity thus this combination therapy increases sensitivity to cancer cells. In addition, treatment with DCA/ADM combination induced significant increase in intracellular ROS levels in the hepatoma cells, suggesting the possible involvement of high intracellular ROS levels in reactions leading to the cytotoxicity increase observed after simultaneous treatment with DCA and ADM. Interestingly, coincubation with 10 mmol/L NAC restored ROS to basal levels accompanied with inhibition of the enhanced cytotoxicity observed with DCA/ADM combination. In addition, exposure to 1 mmol/L BSO (GSH depletion) caused further increase in intracellular ROS levels as well as cell death in comparison with cells treated with the combination alone. Taken together, these data demonstrate that the enhanced cytotoxicity of DCA/ADM combination is mediated by its capacity to induce metabolic oxidative stress.

ADM is known to intercalate between base pairs in the DNA helix, thereby preventing DNA replication and ultimately inhibiting protein synthesis. Additionally, ADM forms free radicals, resulting in cytotoxicity due to peroxidation of cell membrane lipids. It is admitted that accumulation of hydrogen peroxide is an early and crucial step for ADM, Cisplatin, and Paclitaxel induced cancer cell death both *in vitro* and *in vivo* [36]–[38]. Of note, Friesen *et al*. [39] reported that ADM resistant cells exhibited higher GSH levels in comparison with chemosensitive cells. Furthermore, it was shown that downregulation of intracellular GSH reversed deficient drug-induced hyperproduction of ROS and ADM-induced apoptosis [39].

Increase in ROS generation may trigger redox adaptation in cancer cells, leading to upregulation of antioxidant capacity and a shift in redox homeostasis (high ROS generation and elimination) to maintain the ROS levels below the toxic threshold. As such, a moderate increase in ROS amounts induced by exogenous oxidative stress stimuli (e.g. ADM) may not cause elevation of ROS above the threshold. Provided high oxidative stress levels induced by exogenous ROS-generating agents or antioxidant system inhibitory compounds (e.g. DCA) beforehand, a subsequent exogenous ROS stressor (e.g. ADM) is likely to cause elevation of ROS above the threshold level, therefore triggering cell death. These findings provide a biochemical basis for design of combination therapeutics to effectively exploit ROS-mediated mechanisms in cancer cell killing. Interestingly the results presented here show that the cytotoxic behavior of ADM in normal hepatocytes may not be due to ROS-mediated mechanisms as ROS increased in the cancerous cells but not the LO_2_ cells. So the mechanism of ADM action may be different in the different cell types. DCA has an additive effect with ADM by regulating glucose metabolism and ROS production only in cancer cells but not in normal cells. This needs to be investigated further to be fully explained.

We demonstrated that DCA acts both as a modulator of glucose metabolism and a pro-oxidant agent in hepatoma cells. Since most cancer cells exhibit increased aerobic glycolysis and high ROS levels, anticancer strategies based on DCA might achieve promising therapeutics with negligible toxic side effects. Indeed, DCA has been shown to display relatively modest toxicity in extensive clinical trials for lactic acidosis. Interestingly, we found that DCA induced apoptosis and enhanced ADM cytotoxicity in hepatoma cells with no significant effects on normal hepatocytes. This is of high significance as excessive ROS levels are toxic to cells and some organs such as heart, kidney and liver are highly vulnerable to oxidative toxicity [40]. Unlike DCA/ADM combination, we found an increase in intracellular ROS levels and cytotoxicity in LO_2_ cells after treatment with the BSO/ADM combination.

Combining relatively nontoxic drugs such as DCA and ADM might allow enhanced tumor cell killing at lower doses, therefore minimizing ADM side effects as well as overcoming potential resistance to ADM.

In conclusion, small-molecule DCA significantly enhances the ADM cytotoxicity in hepatoma cells via elevated ROS generation without affecting noncancerous liver cells. Thus, these data provide a strong biochemical rationale for the use of DCA in combination with conventional chemotherapeutic drugs for cancer therapy in clinical settings.
